# Development and Validation of a Radiomic Nomogram for Predicting the Prognosis of Kidney Renal Clear Cell Carcinoma

**DOI:** 10.3389/fonc.2021.613668

**Published:** 2021-07-06

**Authors:** Ruizhi Gao, Hui Qin, Peng Lin, Chenjun Ma, Chengyang Li, Rong Wen, Jing Huang, Da Wan, Dongyue Wen, Yiqiong Liang, Jiang Huang, Xin Li, Xinrong Wang, Gang Chen, Yun He, Hong Yang

**Affiliations:** ^1^ Department of Medical Ultrasound, The First Affiliated Hospital of Guangxi Medical University, Nanning, China; ^2^ Department of Urology, The First Affiliated Hospital of Guangxi Medical University, Nanning, China; ^3^ Department of Radiology, The First Affiliated Hospital of Guangxi Medical University, Nanning, China; ^4^ GE Healthcare Global Research, GE, Shanghai, China; ^5^ Department of Pathology, The First Affiliated Hospital of Guangxi Medical University, Nanning, China

**Keywords:** contrast-enhanced computed tomography, artificial intelligence, kidney renal clear cell carcinoma, prognosis, weighted correlation network analysis

## Abstract

**Purpose:**

The present study aims to comprehensively investigate the prognostic value of a radiomic nomogram that integrates contrast-enhanced computed tomography (CECT) radiomic signature and clinicopathological parameters in kidney renal clear cell carcinoma (KIRC).

**Methods:**

A total of 136 and 78 KIRC patients from the training and validation cohorts were included in the retrospective study. The intraclass correlation coefficient (ICC) was used to assess reproducibility of radiomic feature extraction. Univariate Cox analysis and least absolute shrinkage and selection operator (LASSO) as well as multivariate Cox analysis were utilized to construct radiomic signature and clinical signature in the training cohort. A prognostic nomogram was established containing a radiomic signature and clinicopathological parameters by using a multivariate Cox analysis. The predictive ability of the nomogram [relative operating characteristic curve (ROC), concordance index (C-index), Hosmer–Lemeshow test, and calibration curve] was evaluated in the training cohort and validated in the validation cohort. Patients were split into high- and low-risk groups, and the Kaplan–Meier (KM) method was conducted to identify the forecasting ability of the established models. In addition, genes related with the radiomic risk score were determined by weighted correlation network analysis (WGCNA) and were used to conduct functional analysis.

**Results:**

A total of 2,944 radiomic features were acquired from the tumor volumes of interest (VOIs) of CECT images. The radiomic signature, including ten selected features, and the clinical signature, including three selected clinical variables, showed good performance in the training and validation cohorts [area under the curve (AUC), 0.897 and 0.712 for the radiomic signature; 0.827 and 0.822 for the clinical signature, respectively]. The radiomic prognostic nomogram showed favorable performance and calibration in the training cohort (AUC, 0.896, C-index, 0.846), which was verified in the validation cohort (AUC, 0.768). KM curves indicated that the progression-free interval (PFI) time was dramatically shorter in the high-risk group than in the low-risk group. The functional analysis indicated that radiomic signature was significantly associated with T cell activation.

**Conclusions:**

The nomogram combined with CECT radiomic and clinicopathological signatures exhibits excellent power in predicting the PFI of KIRC patients, which may aid in clinical management and prognostic evaluation of cancer patients.

## Introduction

Regarded as one of the most prevalent tumors of the urogenital system, renal cell cancer (RCC) is a highly malignant cancer derived from the renal epithelium of the parenchyma. In 2020, 45,520 new cases were diagnosed in males and 28,230 in females. RCC accounted for 5% of all male malignancies and 3% of all female malignancies in 2020 (1). KIRC, the most epidemic histological subtype of primary RCC, accounts for almost 90% of all kidney malignancies with five-year survival rates of approximately 44–69% (2, 3). Progress has been achieved through multiple optional methods in surgical resection and systemic therapies for KIRC; however, overall survival and prognosis, especially if the cancer is detected at an advanced stage, are still unsatisfactory if the cancer is not treated optimally, due to high invasiveness, high mortality, and resistance to chemoradiotherapy (2, 4). Worse still, the incidence of RCC has been steadily increasing over the past several years (1, 2). The ability to predict prognosis preoperatively and non-invasively is vital. However, specific biomarkers are still lacking because of the complexity of disease progression and high heterogeneity of KIRC. It is urgent that we explore biomarkers that are capable of predicting and monitoring prognosis with good accuracy and then provide a personalized strategy for judgment of clinical treatment.

Radiomics, as a rapidly developing field of transforming medical images into available data in radiology, has the capability to investigate efficacy monitoring, prognosis surveillance, micro-environment evaluation, and biological behavior assessment *via* quantitatively extracting features and excavating in-depth characterization of tumor phenotypes beyond imaging interpretation (5, 6). Radiomics not only can show relationships between radiomic signatures and genomics, metabolomics, and proteomics but also offer a non-invasive way to create objectively quantitative biomarkers of tumor biology that might be of value in predicting prognosis and therapy response (7). Recently, increasing attention has been focused on the application of computed tomography (CT) radiomic in RCC, which has satisfactory potential in lesion characterization (8–10), histological grade (11–13) and assessment of response to treatment (14, 15). Nevertheless, the correlation between radiomic features and the prognosis of KIRC patients is still undefined, and thorough research should be conducted to provide references for clinical work.

To address the need for a non-invasive, preoperative method of assessing the prognosis of KIRC patients, we have developed a contrast-enhanced computed tomography (CECT) radiomic prognostic signature based on three-dimensional (3D) medical images, and we have identified clinical signature based on clinical parameters in this study. With the combination of radiomic features and clinical parameters, a comprehensive nomogram was established to evaluate the progression-free interval (PFI) of patients suffering from KIRC. In order to further investigate the relationship between radiomic characteristics and gene regulation, weighted correlation network analysis (WGCNA) and function enrichment as well as signaling pathway analysis were performed. Fortunately, the results of this research indicated that our radiomic nomogram could not only predict prognosis and guide clinical therapy of KIRC but also elucidate the underlying molecular mechanism of KIRC.

## Materials And Methods

### Sample Collection

A total of 136 patients with KIRC were collected from our hospital from 2012 to 2016 as the training cohort of the study. This study was approved by the hospital ethics committee, and informed consent was waived due to its retrospective nature. The inclusion criteria were as follows:  (1) KIRC was histologically confirmed postoperatively; (2) patients preoperatively received CECT examination; and (3) CECT images and corresponding prognostic data could be obtained. The exclusion criteria were as follows (1): the patients received preoperative chemotherapy or chemoradiotherapy; (2) the renal lesion was poorly displayed on the images; and (3) preoperative CECT image, relevant clinicopathological parameters of patients were lacking. Data of clinicopathological parameters [age, gender, clinical staging (cTNM), and pathology grade, PFI time] and CECT images were obtained from electronic patient record system.

The validation cohort comprised CECT images of patients with KIRC from The Cancer Imaging Archive (TCIA; http://www.cancerimagingarchive.net/) datasets and their relevant clinicopathological data gathered from websites from The Cancer Genome Atlas (TCGA; https://cancergenome.nih.gov/). The inclusion and exclusion criteria and collection of clinicopathological parameters were consistent with those mentioned above.

### Image Acquisition and Delineation of the Area of Interest

An abdominal CECT examination containing phase scanning of the corticomedullary phases (CMP) (30–40 s), nephrographic phases (NP) (70–90 s), and excretory phases (EP) (3–4 min) was preoperatively adopted in enrolled patients. Three CT scanning instruments were applied in this study, and the specific models and scanning parameter configurations were shown in **Table 1**. In our study, only the corticomedullary phase (CMP) of CECT was used for radiomic analysis, and the identification of CMP was determined by the method of previous studies (16, 17). The 3D volumes of interest (VOI), including the target lesion on the CMP of the CECT images, was segmented by two experienced radiologists with 10 years of radiology experience using ITK-SNAP (http://www.itksnap.org/) (18).

**Table 1:** Summary of parameters of CT models and scanning protocols.

	CT Instruments	Tube Voltage	Tube Current	Slice Thickness	Matrix
**Training cohort**	GE, SIEMENS	100–120 KV	76–659 mA	1–5 mm	512 × 512 matrix
**Validation cohort**	GE, SIEMENS, Philips	120–140 KV	72–620 mA	1.25–5 mm	512 × 512 matrix

GE, GE Healthcare; SIEMENS, SIEMENS Healthcare; Philips, Philips Healthcare.

### Radiomic Features Extraction

Feature extraction was conducted by Ultrasomics (Version 2.1), which is software capable of high-throughput extraction of massive image features (19–21). A total of 2,944 high-throughput radiomic characteristics were acquired automatically from VOI based on each target lesion of the tumor. The radiomic features consisted of six different feature types (1): 122 original (such as first-order statistics, shape descriptors, texture classes, gray-level co-occurrence matrix, gray-level run length matrix, and gray-level size zone matrix) (2); 1,170 co-occurrence of local anisotropic gradient orientations (CoLIAGe) (3); 432 wavelets + local binary pattern (LBP) (4); 1,080 Gabors (5); 80 phased congruency-based local binary pattern (PLBP); and (6) 60 wavelet-based improved local binary pattern (WILBP) features.

### Intraclass Correlation Coefficient Analysis

In order to assess the reproducibility of radiomic features exaction, 30 cases were randomly chosen from all patients, and their CECT images were segmented by the two radiologists mentioned above in a double-blind condition to test the consistency of the delineation of the tumor VOI. The intraclass correlation coefficient (ICC) was adopted to measure the inter-observer consistency of the feature extraction. Radiomic features with ICC values ≥0.75 indicate a strong consistency.

### Sample Grouping and Feature Preprocessing

In the design of this study, the cases from our hospital were used as the training cohort, and the cases from TCIA were used as the validation cohort. Similarly, according to the grouping information, the corresponding radiomic features and clinical parameters were divided into two groups. A calculative model was applied to the training cohort to learn underlying patterns hidden in the datasets, and a validation cohort was used to evaluate the predictability of the model. For these radiomic features, z-score standardization was conducted to normalize the radiomic profiles in the training cohort and validation cohort, respectively.

### Survival Analysis and Establishment of Prognostic Signatures

To search for radiomic features and clinical parameters significantly associated with survival, survival analysis of the training cohort was performed using the “survival” package. We defined PFI as an end point event, and PFI is commonly used in cancer therapy monitoring. The definition of the endpoint was consistent with previous studies (22, 23). Univariate Cox regression analysis was conducted to investigate the relevance of each radiomic feature, clinical variable, and PFI. Significant (*P* < 0.1) variables were contained in the subsequent regressive analysis. Aimed at selecting predictors with the highest predictive power, using the R “glmnet” package the LASSO-penalized Cox regression algorithm was adopted to reduce the dimension of high-dimensional data in the training cohort and to select the radiomic features with the strongest prognostic value and the lowest relationship among each other (24, 25). In LASSO regression, the optimal Lambda value was chosen according to the minimum mean square error. With the help of the “survival” package, multivariable Cox analysis was applied to further determine the most useful prognostic radiomic features and clinical variables with independent prognostic values using stepwise regression analysis and the best subset regression method. Subsequently, a radiomic signature and a clinical signature were established by linear combination method. The weight coefficients of the radiomic features and clinical variables were derived from the regression coefficients in multivariate survival analysis by setting the PFI as the attributive variable. The KIRC patients were split into low- and high-risk groups according to the median risk score of each risk signature. The KM curve, time-dependent ROC curve, and Concordance index (C-index) were used to assess the efficiency of each risk signature by using the “survivalROC” package and the “survcomp” package (26, 27).

### Development and Validation of the Nomogram

To explore the prognostic value of the combinative signature with clinical factors, we took the radiomic signature and meaningful clinical parameters into the Cox regression model to generate a combined clinical–radiomic model. In order to visualize model efficiency, the trained cohort was applied to develop the easy-to-use nomogram of the clinical prognostic prediction model using the “rms” package, and the validated cohort was used for external verification. Similarly, high- and low-risk groups were determined based on the median risk score from the clinical–radiomic prediction model, and KM curves were drawn to assess differences in PFI between the two groups of patients. C-index and ROC curve analysis were used to measure nomogram performance. A calibration curve was utilized to assess the predictive accuracy of the nomogram, and model fitness was assessed by the Hosmer and Lemeshow goodness-of-fit test.

### WGCNA and Functional Analysis

In order to investigate the molecular microcosmic meaning of radiomic features and reveal the underlying association of radiomic features and transcriptome molecular function, unsigned WGCNA was performed to determine genes that were correlated to prognostic radiomic features using the “WGCNA” package (28). WGCNA is a systematic biology approach illustrating patterns of gene relevance of different phenotypes and seeking clusters (modules) of highly relevant genes and correlative modules with external sample traits. Transcriptomics data of KIRC were acquired from TCGA. In this study, only the protein-encoding messenger RNAs (mRNAs) were selected to investigate the molecular functional characteristics of KIRC; low-abundance protein-coding genes with average log2 (count + 1) values <0.5 were discarded. The gene modules that correlated with radiomic features most significantly were selected as the key modules, which were used for subsequent function enrichment and signaling pathways analysis. The “clusterProfiler” package was used for performing Gene Ontology (GO) and Kyoto Encyclopedia of Genes and Genomes (KEGG) analysis. As a conventional method, GO enrichment analysis was applied to assess biological process (BP), molecular functions (MF), and cellular components (CC) involved in the genes of interest. KEGG pathway analysis was aimed at identifying the underlying functional and signaling pathways connected to modules genes.

## RESULTS

### Patient Clinical Parameters

**Figure 1:**
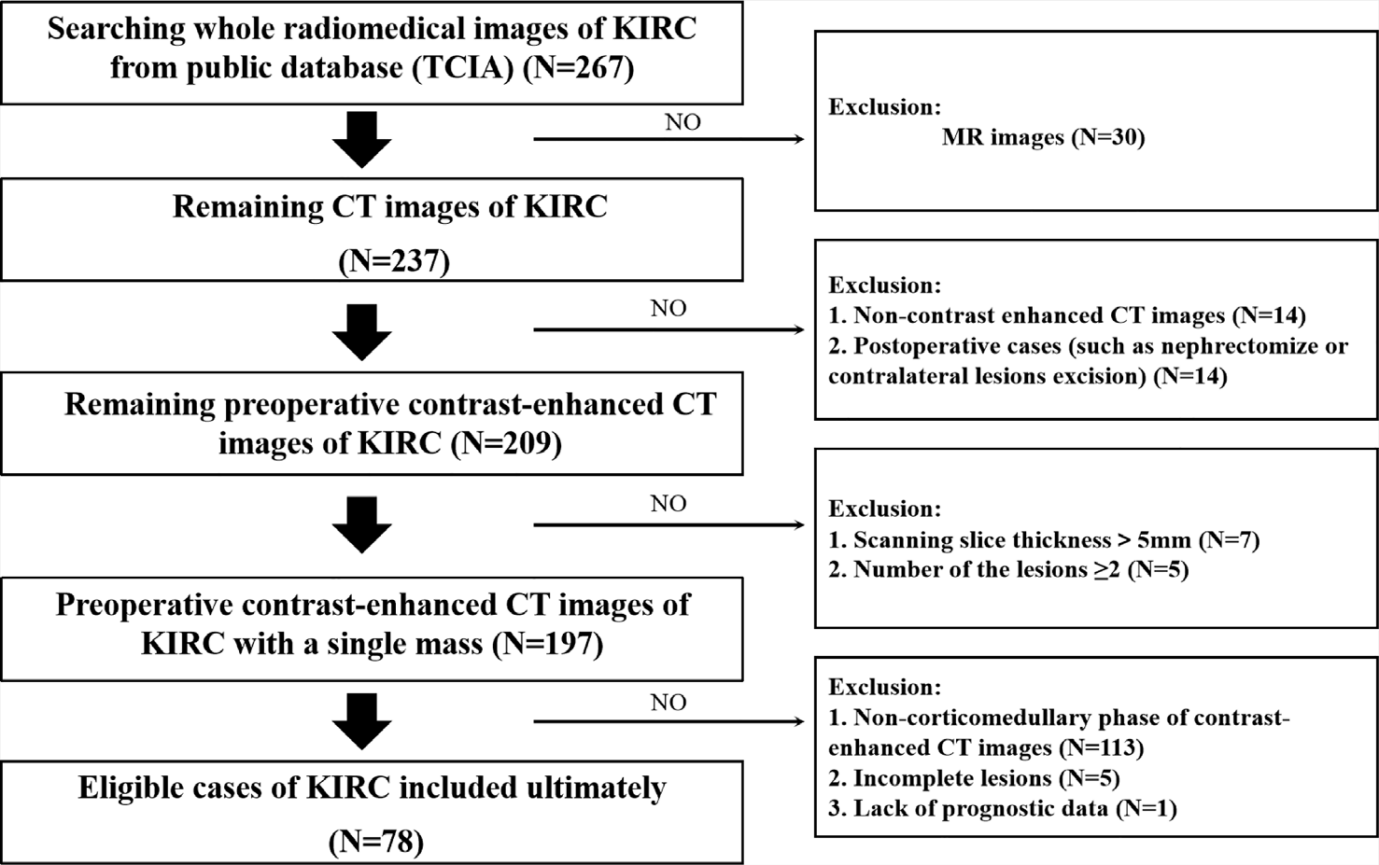
Technology roadmap of this study.

**Figure 2:**
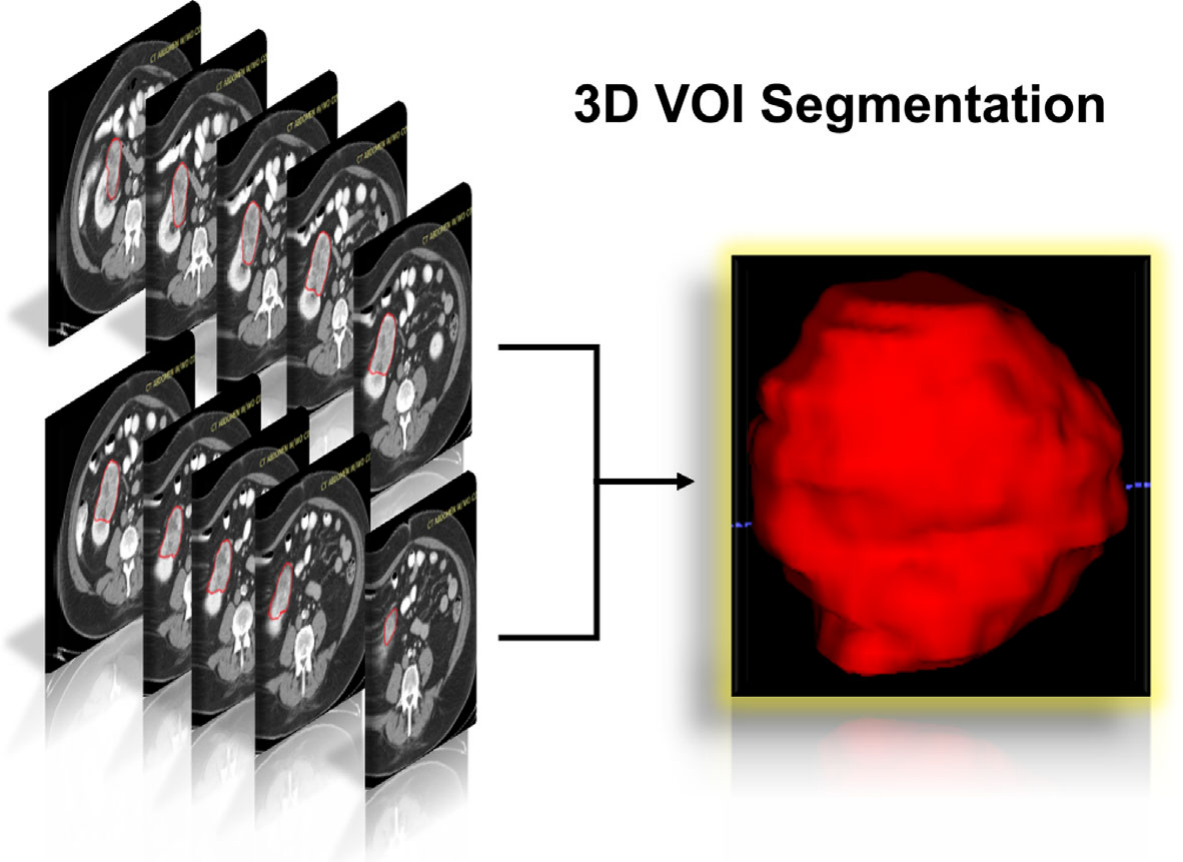
Schematic diagram of the complete 3D geometric image obtained by manually drawing and segmenting the VOI from the CECT examination. *3D, three dimensions; VOI, volumes of interest; CECT, contrast-enhanced computed tomography*.

The flowchart of our research was displayed in **Figure 1**, and the flowchart of patients selected and included from TCIA was shown in **Supplementary Figure 1**. There were 78 KIRC patients from TCGA who satisfied the entry criteria for enrolling in the study. The schematic diagram of the complete 3D geometric image obtained by manually drawing and segmenting the VOI was shown in **Figure 2**. Detailed clinical baseline characteristics of patients were presented in **Supplementary Table 2**. In the training cohort, there were 97 cases in males and 39 cases in females, with a median age of 53 years and an age range of 20–81 years. The median follow-up time for PFI was 1,470 days. In the validation cohort, there were 44 cases in males and 34 cases in females, with a median age of 59 years and an age range of 26–85 years. The median follow-up time for PFI was 1,227 days.

### Intraclass Correlation Coefficient Analysis and Feature Preprocessing

A total of 2,944 features were extracted from the CT images. The average and median values of the inter-observer ICC of radiomic features were 0.814 and 0.974. The ICC result showed good consistency between groups. After the ICC analysis, there were 2,244 (76.22%) radiomic features with ICC≥0.75, which indicated that these radiomic features had good reproducibility. Z-score standardization of radiomic features was done as described in the *Materials and Methods* section. A total of 1,901 radiomic features were used for subsequent prognostic analysis.

### Radiomic Features and Clinical Variable Selection

**Figure 3:**
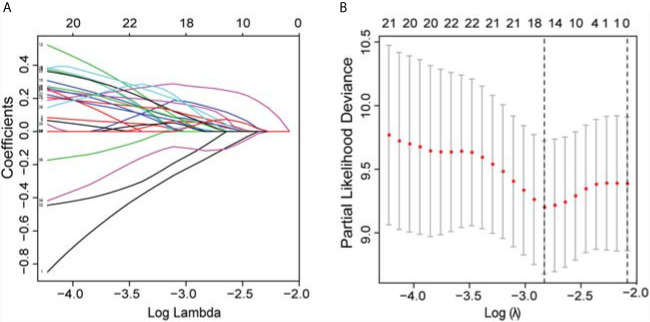
LASSO was utilized to identify the radiomic features that highly correlated with the PFI of KIRC patients. **(A)** LASSO path map, radiomic features corresponding to different alpha features. **(B)** Optimal lambda resulted in 16 non-zero coefficients for the radiomic signature. *PFI, progression-free interval; LASSO, least absolute shrinkage and selection operator; KIRC, Kidney Renal Clear Cell Carcinoma*.

In the univariate Cox regression analyses, 43 radiomic features, age, cTNM, and grade were significantly correlated with the PFI of KIRC patients (*P* < 0.1) and were used for subsequent investigation. In multivariate analysis, double feature-dimension reduction methods (LASSO and stepwise regression analysis) were used to identify the 10 radiomic features and clinical variable (cTNM) (*P* < 0.05) that were independent prognostic markers for PFI (**Figure 3**). Although age (*P* = 0.051) and pathological grade (*P* = 0.059) did not show significant significance in multivariate Cox regression analysis, both of them are clinically important factors affecting the prognosis of KIRC, so we also included them in our clinical prediction signature (**Table 2** **)**.

**Table 2:** Univariate and multivariate Cox regression analyses of clinicopathological parameters.

Univariate Cox Regression				Multivariate Cox Regression		
Variable	B	P	HR (95% CI)	B	P	HR (95% CI)
Gender	−0.267	0.517	0.766 (0.341–1.718)			
Age	0.030	0.060	1.030 (0.999–1.063)	0.033	0.051	1.033 (1.000–1.068)
cTNM	1.883	<0.001*	6.576 (3.014–14.347)	1.593	<0.001*	4.916 (2.151–11.237)
Grade	1.062	0.009*	2.891(1.310–6.378)	0.810	0.059	2.249 -(0.969–5.218)

HR, hazard ratio; *p < 0.05.

### Development and Validation of the Prognostic Signatures

**Figure 4:**
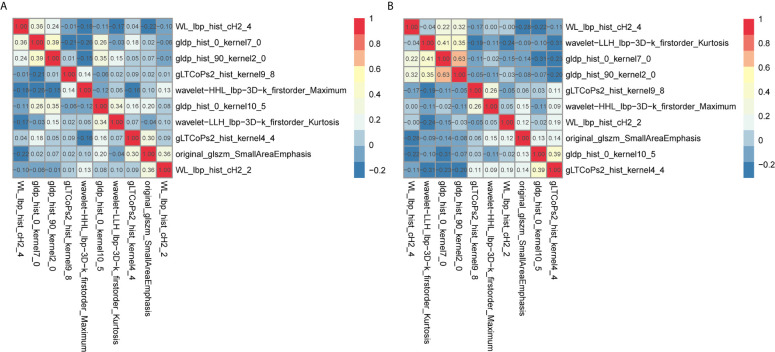
Co-expression heat maps of radiomic features used to build the radiomic signature. **(A, B)** co-expression heat maps of the radiomic modeling features in the training cohort and validation cohort. Positive correlation represents co-expression relationships between radiomic features; and negative correlation represents negative co-expression relationships between features. Red indicates a positive correlation; blue indicates a negative correlation.

**Figure 5:**
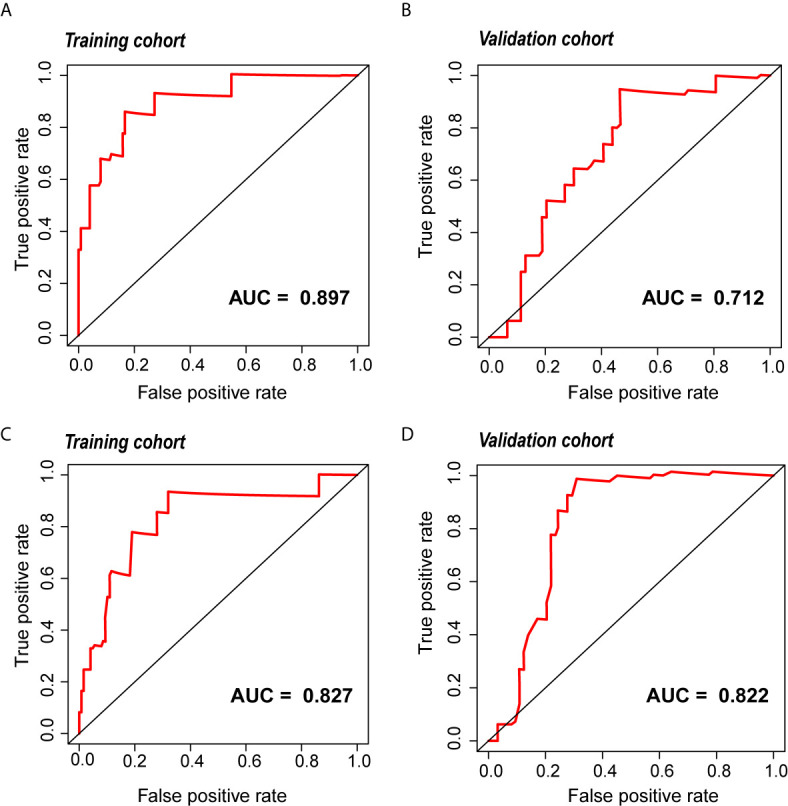
The ROC curve of the radiomic and clinical signature. **(A, B)** The ROC of the radiomic signature in the training cohort and the validation cohort. **(C, D)** The ROC of the clinical signature in the training cohort and the validation cohort. *ROC, relative operating characteristic curve*.

The radiomic prognostic signature, consisting of ten features, and the clinical prognostic signature, consisting of three clinical variables, were constructed by multivariate Cox analysis (**Table 3** and **Supplementary Table 2**). The correlation analysis heat maps of the modeling radiomic features in the training cohort and the validation cohort were shown in **Figure 4**. In terms of prediction accuracy, we found that our prediction signatures performed well. For radiomic signature, the AUC for the training cohort was 0.897, and the AUC for the validation cohort was 0.712 (**Figures 5A, B**). The AUC of the training cohort in the clinical signature was 0.827, and the AUC of the validation cohort was 0.822 (**Figures 5C, D** **)**. The C-index values of the radiomic signature and the clinical signature were 0.861 (95% CI 0.789–0.927) and 0.784 (95% CI 0.696–0.872), respectively. The KM survival curve analysis of both the radiomic signature and the clinical signature revealed that the PFI of the high-risk group was dramatically shorter than that of the low-risk group (*P* < 0.05) (**Supplementary Figures 2** and **3**). These results mean that the above-risk signatures performed well in predicting the clinical outcome of KIRC patients.

**Table 3:** Univariate and multivariate Cox regression analysis of radiomic normogram.

Univariate logistic regression analysis				Multivariate logistic regression analysis		
Variable	B	P	HR (95% CI)	B	P	HR (95% CI)
Gender	-0.267	0.517	0.766 (0.341-1.718)			
Age	0.030	0.060	1.030 (0.999-1.063)	0.036	0.055	1.036 (0.999-1.075)
cTNM	1.883	<0.001*	6.576 (3.014-14.347)	1.131	0.019*	3.099 (1.203-7.984)
Grade	1.062	0.009*	2.891 (1.310-6.378)	0.869	0.047*	2.384 (1.013-5.610)
Radiomic signature	0.014	<0.001*	1.014 (1.009-1.020)	0.012	<0.001*	1.012 (1.006-1.017)

HR, hazard ratio; *p < 0.05.

### Nomogram Construction and Evaluation

**Figure 6:**
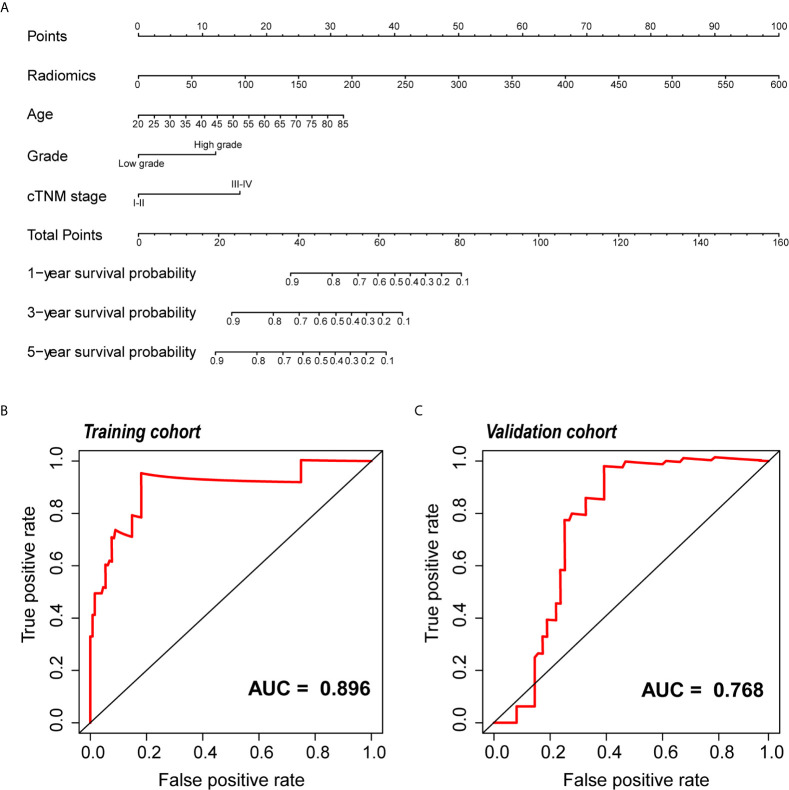
Development and validation of the nomogram. **(A)** The nomogram for predicting 1, 3, and 5 years PFI of KIRC patients. An example of how to use the nomogram was presented below: a patient that has a radiomic score of 150 and is 60 years old; the pathological grade is high and the cTNM stage is IV. According to the point scale on the nomogram, the points for the four indicators are 25, 10, 12.5 and 0, then the points of these four factors are added up to a total score of 47.5. The next step is to find 47.5 points on the total points scale below, and draw a line perpendicular to the following three axes. Then the probability of one-year PFI of this patient is between 0.8 and 0.9, which is about 0.82, indicating that the probability of one-year PFI of this patient is 82%, and the remaining probability values can be obtained in the same way. **(B, C)** ROC curve of the nomogram for predicting PFI in the training and the validation cohorts. *PFI, progression-free interval; KIRC, Kidney Renal Clear Cell Carcinoma; ROC, relative operating characteristic curve*.

**Figure 7:**
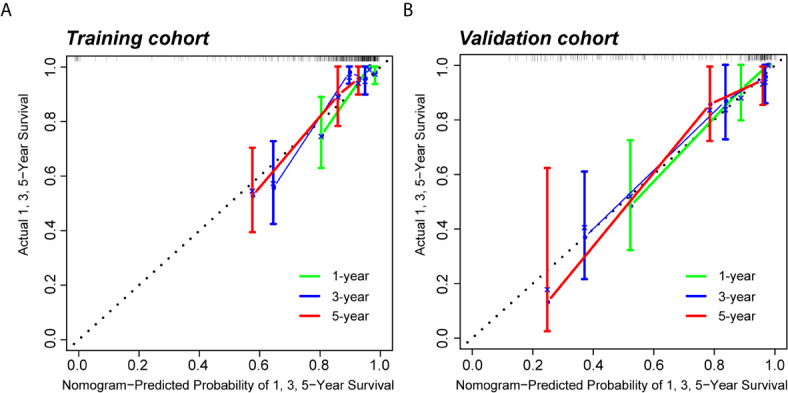
Calibration curves of the predictive nomogram. **(A, B)** Calibration curves of the nomogram to predict the probability of PFI at 1, 3, and 5 years in the training cohort and the validation cohort. *PFI, progression-free interval*.

**Figure 8:**
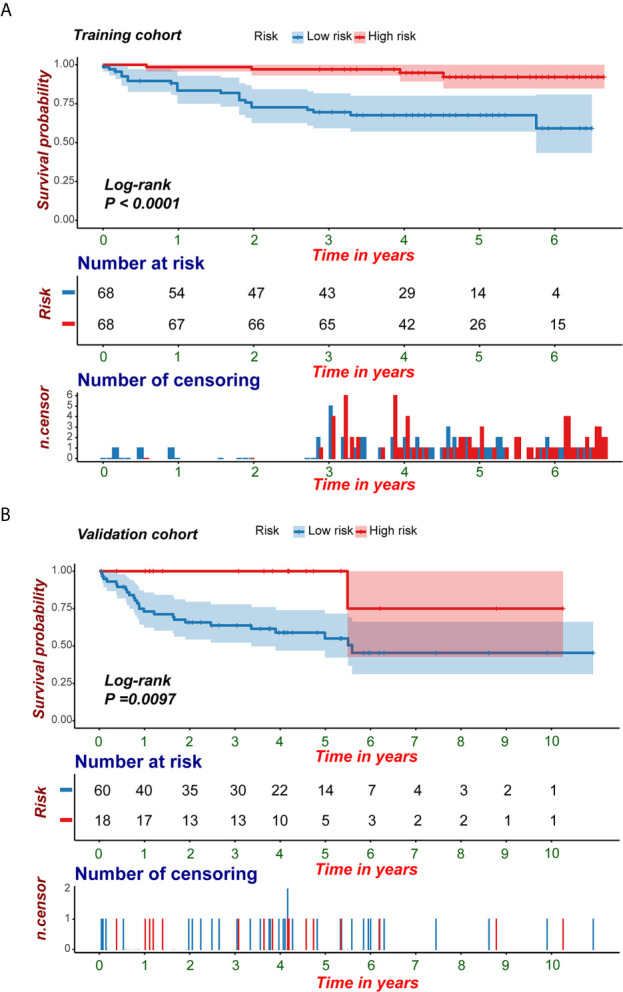
KM survival analyses of the predictive nomogram. **(A, B)** KM analysis of the predictive nomogram indicated that the high-risk group had a shorter PFI compared with that of the low-risk group. *KM, Kaplan-Meier; PFI, progression-free interval*.

Because clinical characteristics are also important factors in cancer outcome, they were added to the comprehensive multivariate Cox regression model. A comprehensive nomogram including radiomic score and clinical pathological parameters was developed and visualized for intuitively predicting the PFI of KIRC patients (**Figure 6A**). The comprehensive risk model in the training cohort had an AUC of 0.896 in predicting PFI of KIRC, and the AUC was 0.768 in the validation cohort (**Figures 6B, C** **)**. The C-index was 0.846. The calibration curves exhibited good agreement between the forecast by the nomogram and actual 1-, 3- and 5-year PFI in both the training cohort and the validation cohort (**Figures 7A, B** **)**. Determination coefficient (R^2^) was used to test the goodness fit of the model. In the present design, the value of the determination coefficient was R^2^ = 0.381. The survival analysis showed that the PFI time of the high-risk group was significantly shorter than that of the low-risk group (**Figure 8**). Collectively, these consequences indicated that the clinical–radiomic signature was a valuable prognostic index for KIRC patients’ stratification and a good indicator for outcome.

### Molecular Characteristics of the Radiomic Features

**Figure 9:**
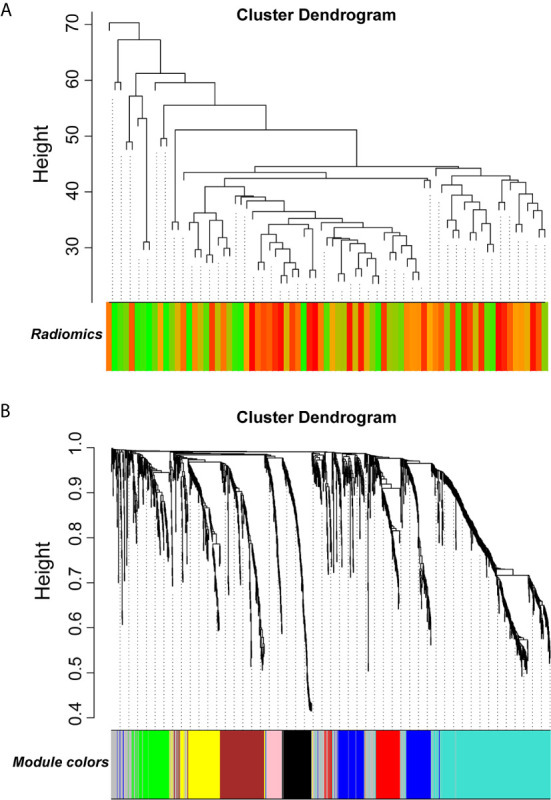
Gene modules associated with radiomic risk scores were determined by WGCNA. **(A)** The association between diversified samples. **(B)** Cluster dendrogram and module assignment for modules from network analysis. *WGCNA, weighted correlation network analysis*.

**Figure 10:**
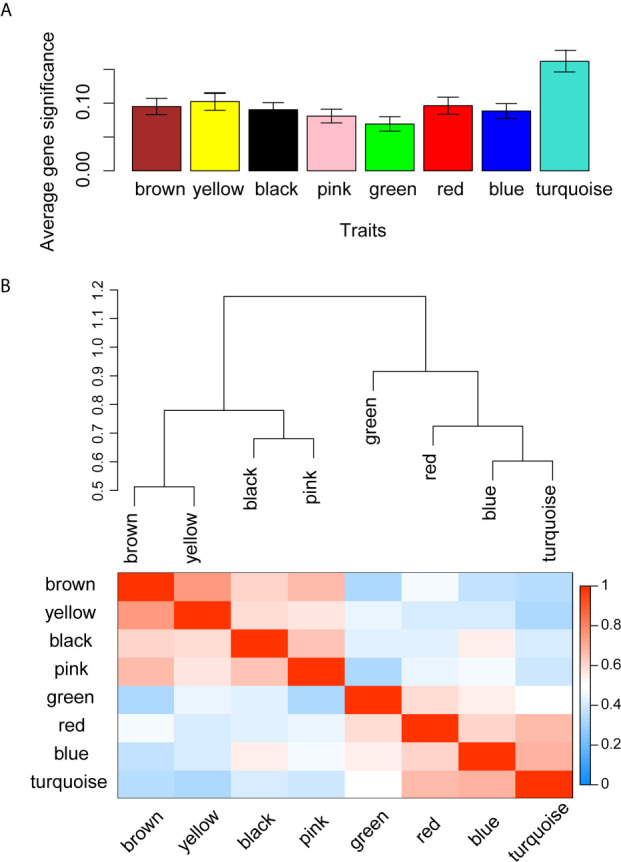
Gene co-expression module identification and correlation analysis. **(A)** Distribution of average gene significance in the modules related with radiomic risk scores. The y-axis represents the significance values. **(B)** The heat map of the correlation between gene modules.

**Figure 11:**
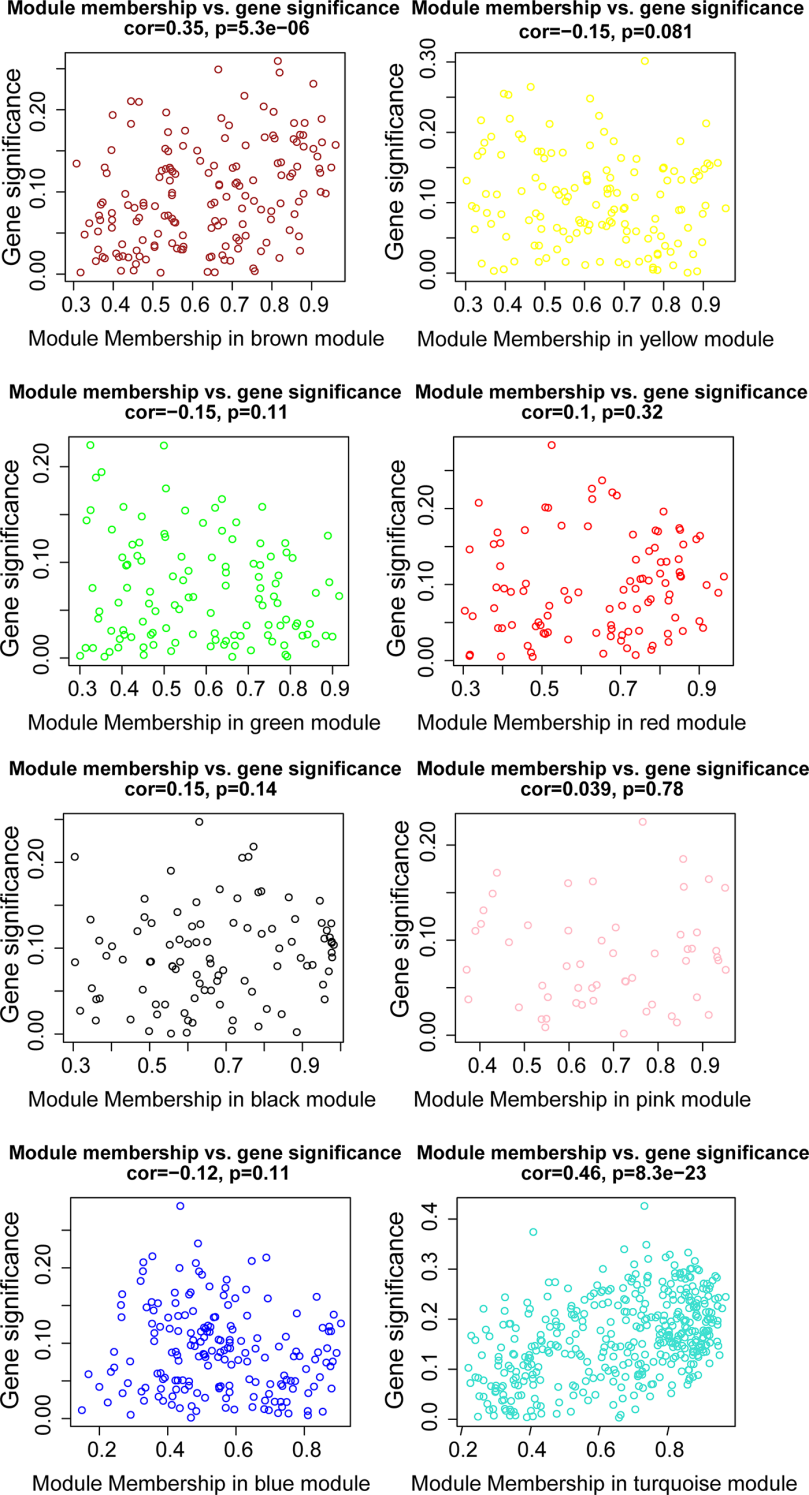
The relationships between the radiomic signature and genes in eight modules. The turquoise modules was highly associated with radiomic risk score and the genes that were selected for further analysis.

**Figure 12:**
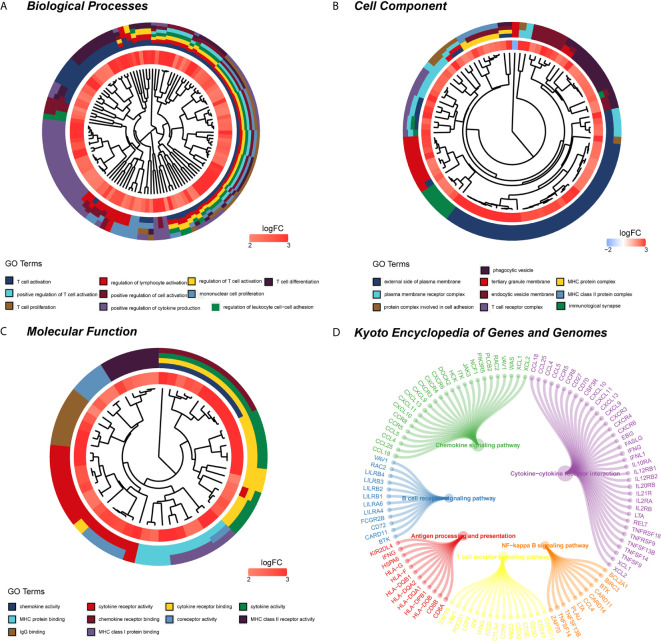
Functional enrichment analysis and signaling pathway analyses of genes associated with radiomic signature. **(A)** Biological process. **(B)** Cellular components. **(C)** Molecular functions. **(D)** KEGG pathway. *KEGG, Kyoto Encyclopedia of Genes and Genomes*.

WGCNA was applied to seek highly co-expressed gene modules and to investigate the correlation between modules and biological traits. WGCNA networks are superior to correlation networks because genes could be zoned into various modules, probably with similar biological function within each individual module (29). To investigate the underlying molecular function of radiomic features, WGCNA was used to find gene modules of highly correlated radiomic signature risk scores (**Figures 9A, B** **)**. Eight modules of covariant gene sets were identified to be correlated with radiomic risk score (**Figure 10A**). Correlation analysis between each module was performed and visualized as a correlation heat map (**Figure 10B**). Among these eight modules, the most relevant module is the turquoise module (R = 0.46, *P* = 8.3e-23, **Figure 11**), which was selected for functional enrichment analysis. The functional analysis showed that genes in the turquoise module were most enriched in T cell activation in BP. For CC, genes were most strongly related to immunological synapse. For MF, genes of modules were mainly enriched in chemokine activity. KEGG analysis of those genes showed their enrichment in T cell receptor signaling pathway. The ten most meaningful pathways of these four enrichment analyses were shown in **Figure 12** and **Table 4**.

**Table 4:** GO and KEGG pathway enrichment analysis of radiomic-related genes.

Categories	ID	Description	P. adjust
BP	GO:0042110	T cell activation	2.75E-53
	GO:0051249	regulation of lymphocyte activation	2.71E-41
	GO:0050863	regulation of T cell activation	2.71E-41
	GO:1903037	regulation of leukocyte cell–cell adhesion	8.27E-38
	GO:0050870	positive regulation of T cell activation	5.66E-30
	GO:0050867	positive regulation of cell activation	1.54E-29
	GO:0032943	mononuclear cell proliferation	1.56E-29
	GO:0030217	T cell differentiation	4.41E-28
	GO:0042098	T cell proliferation	3.43E-25
	GO:0001819	positive regulation of cytokine production	3.48E-25
CC	GO:0009897	external side of plasma membrane	1.50E-19
	GO:0070821	tertiary granule membrane	1.60E-08
	GO:0042611	MHC protein complex	1.16E-06
	GO:0001772	immunological synapse	1.42E-06
	GO:0098802	plasma membrane receptor complex	5.60E-06
	GO:0030666	endocytic vesicle membrane	9.12E-06
	GO:0042613	MHC class II protein complex	1.23E-05
	GO:0045335	phagocytic vesicle	1.23E-05
	GO:0098636	protein complex involved in cell adhesion	7.35E-05
	GO:0042101	T cell receptor complex	0.00291
MF	GO:0008009	chemokine activity	2.17E-06
	GO:0004896	cytokine receptor activity	3.23E-06
	GO:0005126	cytokine receptor binding	5.49E-06
	GO:0005125	cytokine activity	5.49E-06
	GO:0042287	MHC protein binding	9.69E-06
	GO:0042379	chemokine receptor binding	9.69E-06
	GO:0015026	coreceptor activity	1.92E-05
	GO:0032395	MHC class II receptor activity	5.76E-05
	GO:0019864	IgG binding	9.22E-05
	GO:0042288	MHC class I protein binding	0.000125
KEGG	hsa04060	Cytokine-cytokine receptor interaction	3.24E-12
	hsa04061	Viral protein interaction with cytokine and cytokine receptor	1.81E-10
	hsa04658	Th1 and Th2 cell differentiation	2.44E-10
	hsa04514	Cell adhesion molecules	5.32E-10
	hsa04062	Chemokine signaling pathway	1.79E-09
	hsa04650	Natural killer cell mediated cytotoxicity	5.78E-09
	hsa04660	T cell receptor signaling pathway	1.52E-06
	hsa04612	Antigen processing and presentation	1.17E-05
	hsa04662	B cell receptor signaling pathway	1.85E-05
	hsa04064	Cytokine-cytokine receptor interaction	3.21E-09

GO, gene ontology; KEGG, Kyoto Encyclopedia of Genes and Genomes; BP, biological process; CC, cellular components; MF, molecular functions.

## Discussion

As we expected, it has been a focus that a combination of radiomic and clinical markers would help predict survival outcome non-invasively and guide clinical decisions for clinicians. Our study is innovative because this is the first time 3D radiomic signature and clinicopathological characteristics of KIRC patients have been comprehensively integrated with CECT to confirm radiomic indicators for predicting the PFI of patients, and it is the first time a nomogram that can visually display numerical quantization of each factor to predict survival of KIRC patients has been developed. We investigated the correlation between radiomic features and molecular biological characteristics, which might be conducive to a deeper understanding of biological processes and molecular mechanisms in KIRC. The excellent performance of our radiomic signature, clinical signature, and predictive nomogram was observed based on our results, and it suggests that our models can be used to efficiently predict prognosis of KIRC patients and create a robust clinical decision framework for clinicians.

As the most common subtype, which comprised almost 90% of RCC patients in clinic, KIRC has strong potential to metastasize, resulting in the worst prognosis (30). Patients diagnosed with KIRC with lymph node involvement or distant metastasis have low five-year survival rates (31). Additionally, KIRC patients with the same type of tumor might have different prognoses due to the complex internal structure and high heterogeneity within tumors. Complete resection or percutaneous core histopathology biopsy is still a traditional invasive method to assess prognostic indicators (*i.e.* histological classification, grades, and stages) of KIRC for guiding further treatment (30, 32). An objective and non-invasive approach is needed to evaluate and predict clinical outcome of patients with KIRC. CECT performs a vital part in the diagnosis and prognosis monitoring of renal disease because it is non-invasive and convenient, especially when compared to biopsy, surgery, and immunohistochemistry. Biopsy is not always necessary, because imaging is a highly accurate way of characterizing renal malignancy (33). Radiomics, which has been a popular way to extract characteristics in mass data from each medical image, could provide the characteristics and functions of tumors at the macroscopic even at micromolecular level (7). Recently, several studies of immense value in exploring the biological progress of KIRC *via* the construction of radiomic models by CT images have been published. Zhan Feng and Burak Kocak B et al., respectively proved that CT radiomic has the potential to predict BRCA1-associated protein 1 (BAP1) mutation status in KIRC patients (34, 35). Payel Ghosh et al. provided a radiomic–genetics pipeline that can extract 3D intra-tumor heterogeneity features from CECT images and explore associations between features and gene mutation status (36). A proposed integrative radiogenomics method could evaluate risk of postoperative metastasis in KIRC with pathological stage T1, which would be beneficial for postsurgical metastasis treatment of KIRC patients (37). Burak Kocak et al. provided a radiomic model to predict histopathologic nuclear grade by using the radiomic features extracted from unenhanced CT texture analysis of KIRC tumors (13). Other researches constructed classification models that preoperatively identified pathological grades of KIRC patients by using machine-learning-based CT radiomic with non-invasion (38–43). Certain studies also showed the significance of CT radiomic in distinguishing KIRC from other renal mass diseases. Ruimeng Yang et al. developed various machine-learning-based classification models to differentiate renal angiomyolipoma and KIRC with favorable performance (44). Heidi Coy et al. illustrated the utility of machine learning in differentiating KIRC from oncocytoma on routine CT images by using their models, which had the ability to accurately predict renal lesion histology on imaging (45). Xiaoli Meng et al. proposed a CT-based radiomic method to distinguish sarcoma and KIRC with good diagnostic performance (46). However, no published studies explore and predict the PFI of KIRC patients *via* construction of CT radiomic. 3D analysis has shown that 3D structures of targeted lesion is more representative of tumor heterogeneity than two-dimension analysis (47). Our study is the first to predict the PFI of KIRC patients by developing CT radiomic models based on 3D CECT images, and our model achieves good predictive efficacy. In the area of radiomic signature, the radiomic features that were selected as relative factors of prognosis in our study might reflect the degree of tumor progression and assist in the evaluation of postoperative disease progression, treatment effect, and prognosis prediction of KIRC patients. In the area of genomic analysis, identification of specific molecular biological characteristics and regulatory mechanism could not only assist in management and surveillance for KIRC patients but also improve the diagnosis, prognosis, and therapeutic strategy choices for KIRC patients (48–51). In this study, we are the first to provide a predictive nomogram that integrates radiomic and clinicopathological characteristics for predicting the PFI of KIRC patients. The results indicate that our models could be a pivotal tool for prognostic surveillance of KIRC.

The highlight of this study was to explore the relationship between biological information analysis and radiomic features in KIRC, which would provide further information to help us understand the underlying mechanisms and lay the foundation for accurate diagnosis, prognostic judgment, and optimal strategy choice of KIRC for clinicians. Interestingly, the radiomic risk score we performed was closely bound up with various cells of the immune system, especially T cell activation in biological processes. For all we know, at the molecular level, the tumor often involves various cells of the immune-system participation, and it is a complex interplay that has many stages and steps related to the tumor microenvironment. The role of regulatory T cells in cancer has gained concern, and regulatory T cells play a vital role in the progression of KIRC in internal and peripheral tissues (52–54). The high percentages of regulatory T cell activation in peripheral blood or tumorous tissues were correlated to low survival rates in kidney cancer (55–57). Hence, timely and appropriate anticancer treatment, especially immunotherapy, should take the dynamics of the immune response in KIRC patients into account. Several recent studies had investigated the relationship between the cellular immunity-activating system and radiomic signatures in cancer management. For instance, Roger Sun et al. used radiomic to evaluate tumor-infiltrating CD8 cells and response to anti-PD-1 or anti-PD-L1 immunotherapy, which offered a novel method for predicting the immune phenotype and inferring clinical results for cancer patients (6). Xujie Gao et al. proposed a CT radiomic feature to assess tumor-infiltrating T cells and predict prognosis of gastric carcinoma (58). These findings possibly reflected the close relationship among radiomic features and cells of the immunity-activating system. The radiomic features could serve as non-invasive predictors of immuno-oncological characteristics, and they may aid in treatment and outcome management of cancer patients. However, assessment of the crucial relationship between the radiomic score we developed and immune-system cell response, especially T cell activation, needs further exploration and verification in future studies.

There were some inevitable limitations to our study. First of all, the sample size was insufficient. Our study contained only 214 KIRC patients, and the performance and efficiency of the predictive signatures were limited. A prospective cohort study with larger sample sets is recommended. Moreover, our conclusion depended on two center institutions, which might limit the scope of its generalizability. A multi-center prospective study is required to validate this predictive model in a larger population in the future. Additionally, our model only explored the tumor regions with imaging and clinicopathological characteristics. To the best of our knowledge, the peripheral tumor also provided the biological information related to prognosis monitoring. We recommend further exploration of this aspect in the future.

Summarily, our results show satisfactory performance of CECT radiomic and clinical signatures in predicting clinical prognosis. Risk stratification with specific risk scores by radiomic signature has been accurately performed, and the predictive nomogram, which comprehensively integrates radiomic and clinical signature, has the capability to effectively predict outcomes for KIRC patients and to facilitate clinical decision-making for clinicians. Multi-center studies with larger samples are needed to validate our models for clinical practice.

## Contribution

Kidney renal clear cell carcinoma (KIRC) has a poor overall survival and prognosis especially in advanced stage due to high invasiveness, high mortality, and insensitivity to chemoradiotherapy. Radiomics, as a rapidly developing field of transforming medical images into available data in radiology, has the capability to investigate efficacy monitoring, prognosis surveillance, and biological behavior assessment *via* quantitatively extracting features and excavating in-depth characterization of tumor phenotypes beyond imaging interpretation. Radiomics is expected to become an intelligent tool for clinical diagnosis, efficacy evaluation, and prognosis prediction of cancer. Contrast-enhanced computed tomography (CECT), as an imaging exam way, was commonly used in clinic to perform a vital part in the diagnosis and prognosis monitoring of renal disease. The present study aims to explore the relationship between radiomic features, clinical parameters, and progression-free interval (PFI) of KIRC. We further developed and validated a radiomic nomogram that integrates CECT radiomic signature and clinical–pathological parameters for predicting the clinical outcome of KIRC. Meanwhile, we also conducted the molecular functional enrichment analysis to reveal the potential molecular mechanism. In our results, our radiomic signature, clinical signature, and radiomic nomogram were proved robust for prognostic prediction in KIRC patients. To some extent, this study may reveal the underlying molecular mechanism in the development and progression of KIRC and may contribute to clinical management and prognostic evaluation of patients with KIRC.

## Data Availability Statement

Publicly available datasets were analyzed in this study. This data can be found here: The Transcriptomes data of KIRC were acquired from The Cancer Genome Atlas (TCGA; https://cancergenome.nih.gov/), and the CECT images of patients with KIRC were downloaded from The Cancer Imaging Archive (TCIA; http://www.cancerimagingarchive.net/).

## Ethics Statement

The studies involving human participants were reviewed and approved by the institutional review board and ethics committee of the First Affiliated Hospital of Guangxi Medical University. The ethics committee waived the requirement of written informed consent for participation.

## Author Contributions

RG and HQ took part in the conception and design of the study. PL, RW, JingH, YL, JiangH, CM, CL, and DyW collected and sorted the data. RG, HQ, DyW, XL, and XW participated in data analysis and interpretation. GC, HY, and YH supervised and revised the manuscript. All authors contributed to the article and approved the submitted version.

## Conflict of Interest

Authors XL and XW were employed by GE, Shanghai.

The remaining author declares that the research was conducted in the absence of any commercial or financial relationships that could be construed as a potential conflict of interest.
