# Supplementary material for: Development and Validation of a Radiomic Nomogram for Predicting the Prognosis of Kidney Renal Clear Cell Carcinoma
Source: Front Oncol. 2021 Jul 6;11:613668. doi: 10.3389/fonc.2021.613668 (PMC8290524; doi:10.3389/fonc.2021.613668)
Supplement: Supplementary file 1 [file DataSheet_1.docx]

Supplementary Material

# Supplementary Figures and Tables

## Supplementary Figures


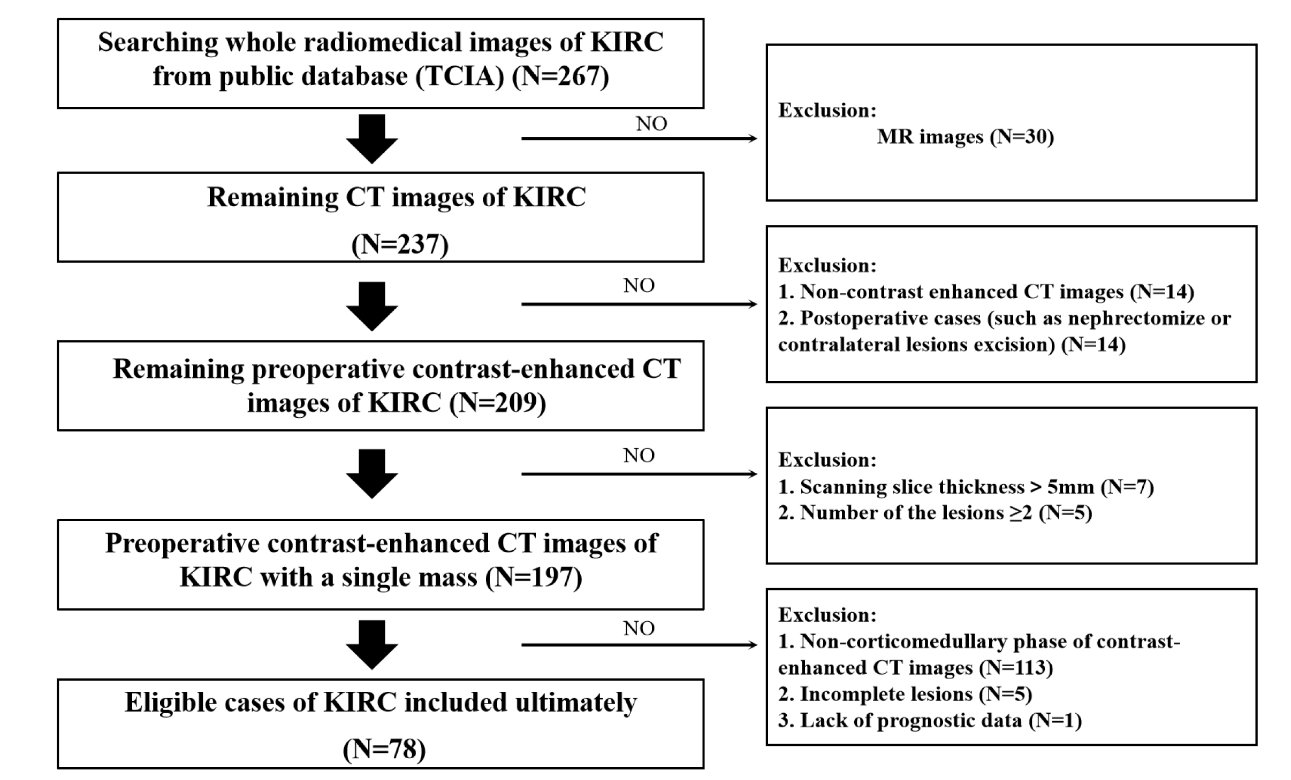


- **Supplementary Figure 1.** The flowchart of patients selected and included from TCIA.


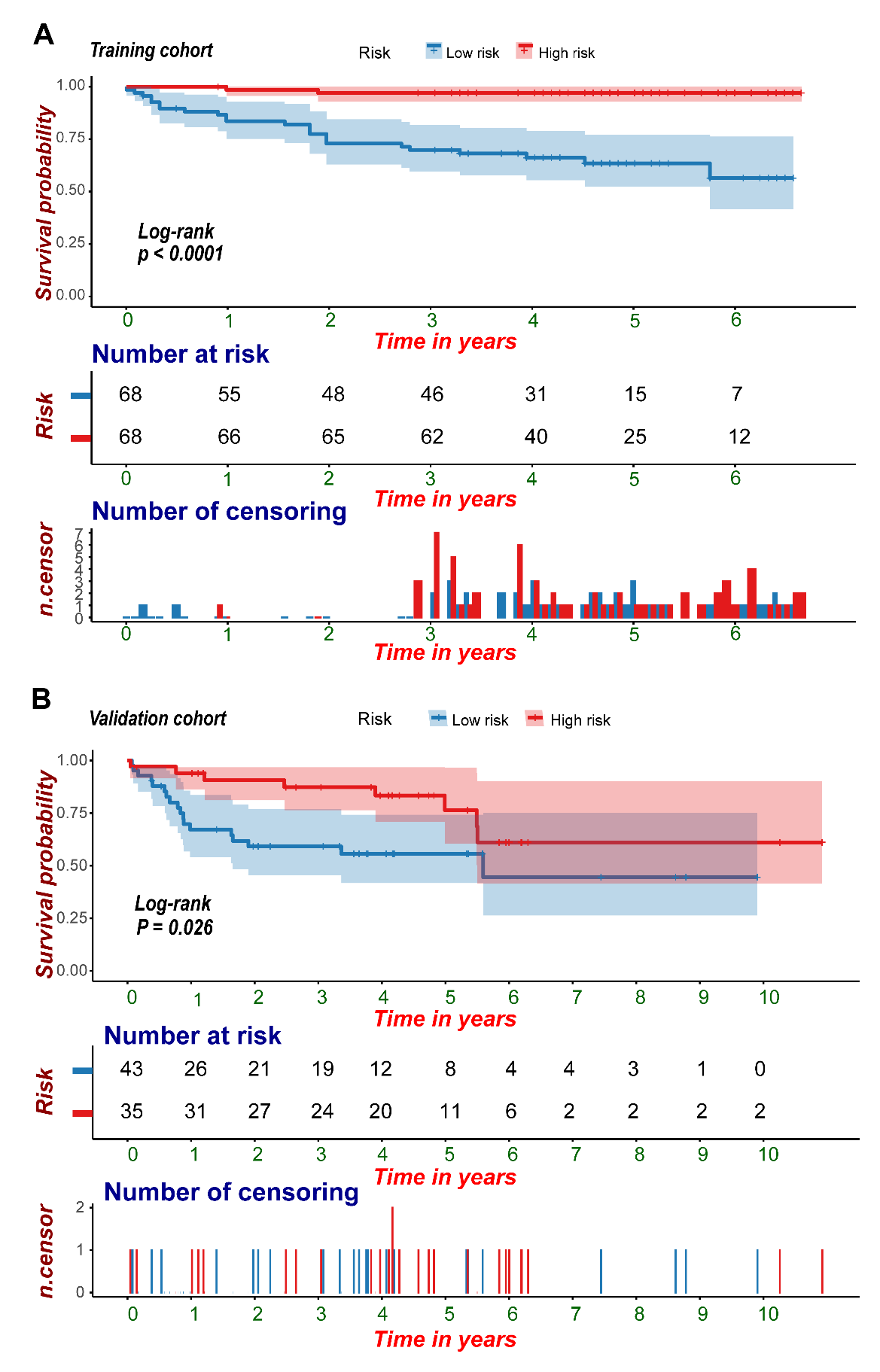


**Supplementary Figure 2.** Patients were split into a high-risk group and a low-risk group according to the median value of the radiomic signature risk score. **(A,B)** KM analysis of the radiomic signature in the training and validation cohort indicated that the high-risk group had a shorter PFI than that of the low-risk group. *KM, Kaplan-Meier; PFI, progression-free interval.*


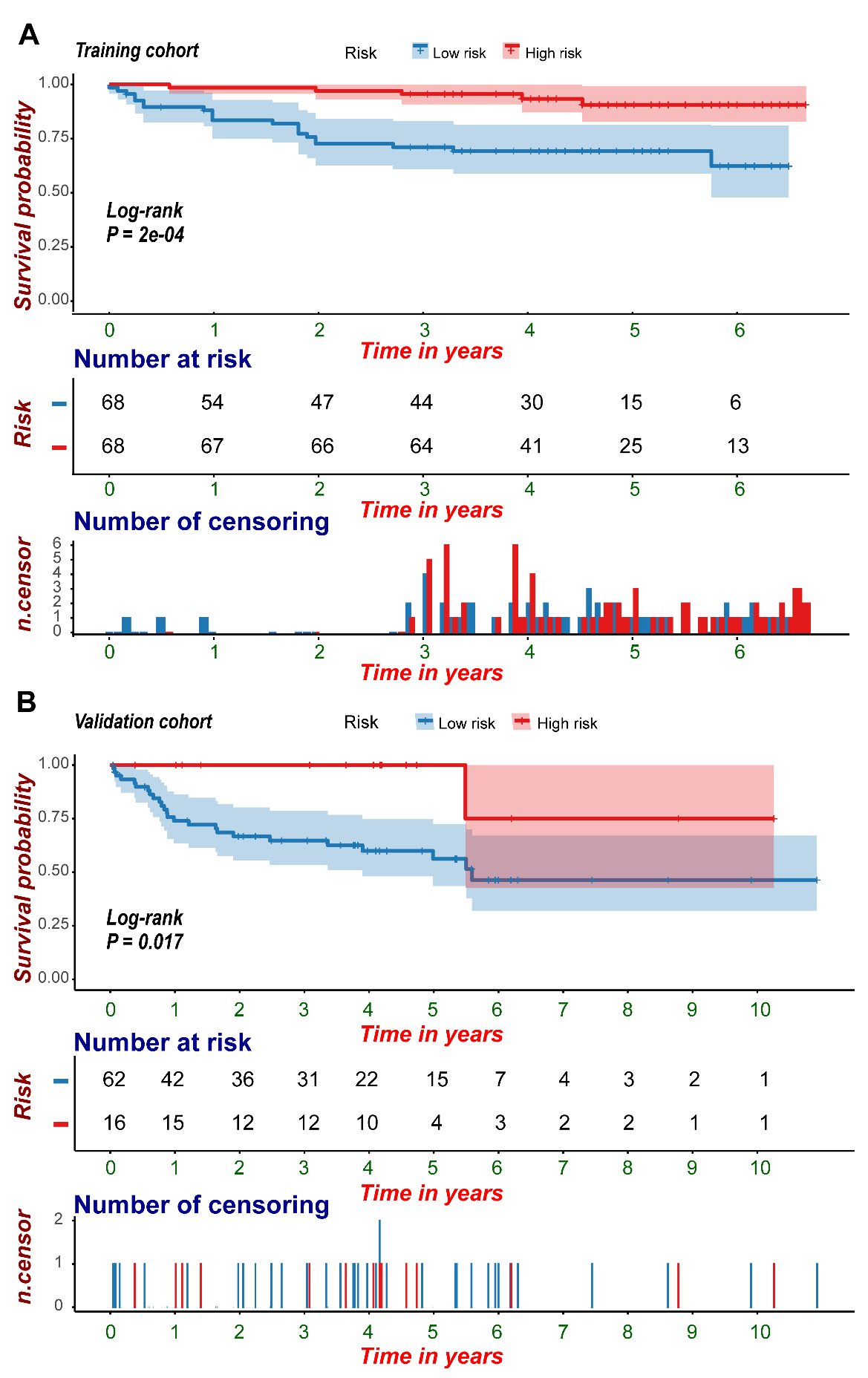


**Supplementary Figure 3.** Patients were split into a high-risk group and a low-risk group according to the median value of the clinical signature risk score. (A,B) KM analysis of the clinical signature in the training and validation cohort indicated that the high-risk group had a shorter PFI than that of the low-risk group. *KM, Kaplan-Meier; PFI, progression-free interval.*

## Supplementary Tables

**Supplementary Table 1** Clinicopathological data of 214 KIRC patients in the training cohort and the validation cohort.

| **Characteristics** | **Training cohort** | **Validation cohort** |
| --- | --- | --- |
| **Patient (N)** | 136 | 78 |
| **Age (Mean ± SD)** | 52.5±12.9 | 60.3±11.7 |
| **The median follow-up time (Day)** | 1470 | 1227 |
| **Gender** |  |  |
| **Male** | 97(71.3%) | 44(56.4%) |
| **Female** | 39(28.7%) | 34(43.6%) |
| **cTNM** |  |  |
| **I-II** | 113(83.1%) | 42(53.8%) |
| **III-IV** | 23(16.9%) | 36(46.2%) |
| **Pathologic Grade** |  |  |
| **Low Grade** | 83(61.0%) | 29(37.2%) |
| **High Grade** | 53(39.0%) | 49(62.8%) |

*KIRC, Kidney Renal Clear Cell Carcinoma; N, number; SD, standard deviation.*

**Supplementary Table 2** Radiomic features set used to build the radiomic signature.

| **[Feature Type](javascript:;)** | **Modeling features** |
| --- | --- |
| Gabor | gldp_hist_0_kernel10_5 |
|  | gldp_hist_0_kernel7_0 |
|  | gldp_hist_90_kernel2_0 |
|  | gLTCoPs2_hist_kernel4_4 |
|  | gLTCoPs2_hist_kernel9_8 |
| Origina | original_glszm_SmallAreaEmphasis |
| Wavelet+LBP | wavelet-HHL_lbp-3D-k_firstorder_Maximum |
|  | wavelet-LLH_lbp-3D-k_firstorder_Kurtosis |
| WILBP | WL_lbp_hist_cH2_2 |
|  | WL_lbp_hist_cH2_4 |
